# Fast and scalable querying of eukaryotic linear motifs with *gget elm*

**DOI:** 10.1093/bioinformatics/btae095

**Published:** 2024-02-20

**Authors:** Laura Luebbert, Chi Hoang, Manjeet Kumar, Lior Pachter

**Affiliations:** Division of Biology and Biological Engineering, California Institute of Technology, Pasadena, CA 91125, United States; California Institute of Technology, Pasadena, CA 91125, United States; Structural and Computational Biology Unit, European Molecular Biology Laboratory (EMBL), 69117 Heidelberg, Germany; Division of Biology and Biological Engineering, California Institute of Technology, Pasadena, CA 91125, United States; Department of Computing and Mathematical Sciences, California Institute of Technology, Pasadena, CA 91125, United States

## Abstract

**Motivation:**

Eukaryotic linear motifs (ELMs), or Short Linear Motifs, are protein interaction modules that play an essential role in cellular processes and signaling networks and are often involved in diseases like cancer. The ELM database is a collection of manually curated motif knowledge from scientific papers. It has become a crucial resource for investigating motif biology and recognizing candidate ELMs in novel amino acid sequences. Users can search amino acid sequences or UniProt Accessions on the ELM resource web interface. However, as with many web services, there are limitations in the swift processing of large-scale queries through the ELM web interface or API calls, and, therefore, integration into protein function analysis pipelines is limited.

**Results:**

To allow swift, large-scale motif analyses on protein sequences using ELMs curated in the ELM database, we have extended the *gget* suite of Python and command line tools with a new module, *gget elm*, which does not rely on the ELM server for efficiently finding candidate ELMs in user-submitted amino acid sequences and UniProt Accessions*. gget elm* increases accessibility to the information stored in the ELM database and allows scalable searches for motif-mediated interaction sites in the amino acid sequences.

**Availability and implementation:**

The manual and source code are available at https://github.com/pachterlab/gget.

## 1 Introduction

Eukaryotic linear motifs (ELMs), also known as Short Linear Motifs, are short stretches of contiguous amino acids, typically 3–15 residues in length, encoding protein–protein interaction sites. They are mainly located in the intrinsically disordered regions (IDRs) of proteins and are typically found to be highly conserved in orthologous proteins. These modules can encode multiple functionalities, which include modification, degradation, docking, targeting, and binding sites for protein domains. As such, ELM-mediated interactions play an essential role in cellular processes and signaling networks, including the regulation of homeostasis, apoptosis, and differentiation (Davey *et al.* 2012, Van Roey *et al.* 2014). Pathogens like SARS-CoV-2 mimic ELMs to gain entry into the cell (Kruse *et al.* 2021, Mészáros *et al.* 2021), and mutations in sequences containing ELMs contribute to diseases like cancer (Uyar *et al.* 2014, Mészáros *et al.* 2017). As a result, ELM-mediated protein interactions are potential targets for therapeutic intervention (Mészáros *et al.* 2021, Fasano *et al.* 2022, Simonetti *et al.* 2023).

The freely accessible ELM resource has two main components: an exploratory candidate motif search web interface and a database with manually curated linear motif knowledge, including information on binding partners and recognition features along with associated biological context. The database information is derived from the scientific literature by expert ELM curators who analyze motif-containing sequences to capture key insights, such as the residues involved in the interaction, their evolutionary conservation, local sequence context in flanking regions, features of the binding site on the interacting partner, and other motif-related insights. In addition, the curation process captures relevant information on the contextual knowledge, which includes cellular function, location, and taxonomic distribution of motif-containing proteins. Since the database was first created (Puntervoll *et al.* 2003, Dinkel *et al.* 2011), it has been continuously updated and has been widely used for both biomedical studies as well as interactomics, proteomics, and molecular research studies (Carberry 2008, Zhang *et al.* 2012, Dinkel *et al.* 2015, Gouw *et al.* 2018, Kumar *et al.* 2020, 2022, 2023, Benz *et al.* 2022, Gogl *et al.* 2022, Reys and Labesse 2022). Users can search amino acid sequences or UniProt Accessions on the ELM database web interface (http://elm.eu.org/) or by submitting an API request through the ELM server. However, these methods have processing limitations when performing large-scale queries, and many requests being submitted simultaneously can lead to server overload and extended wait times.

To expedite the investigation of ELMs, we have extended the *gget* suite of Python and command line tools (Luebbert and Pachter 2023) with a new module, which efficiently finds ELMs in user-submitted amino acid sequences or UniProt Accessions: *gget elm. gget elm* increases accessibility to the information stored in the ELM database and allows scalable searches for ELMs in amino acid sequences. The command line interface and optional JSON formatted output allow swift integration into existing protein analysis workflows.

## 2 Description

**Figure 2.**
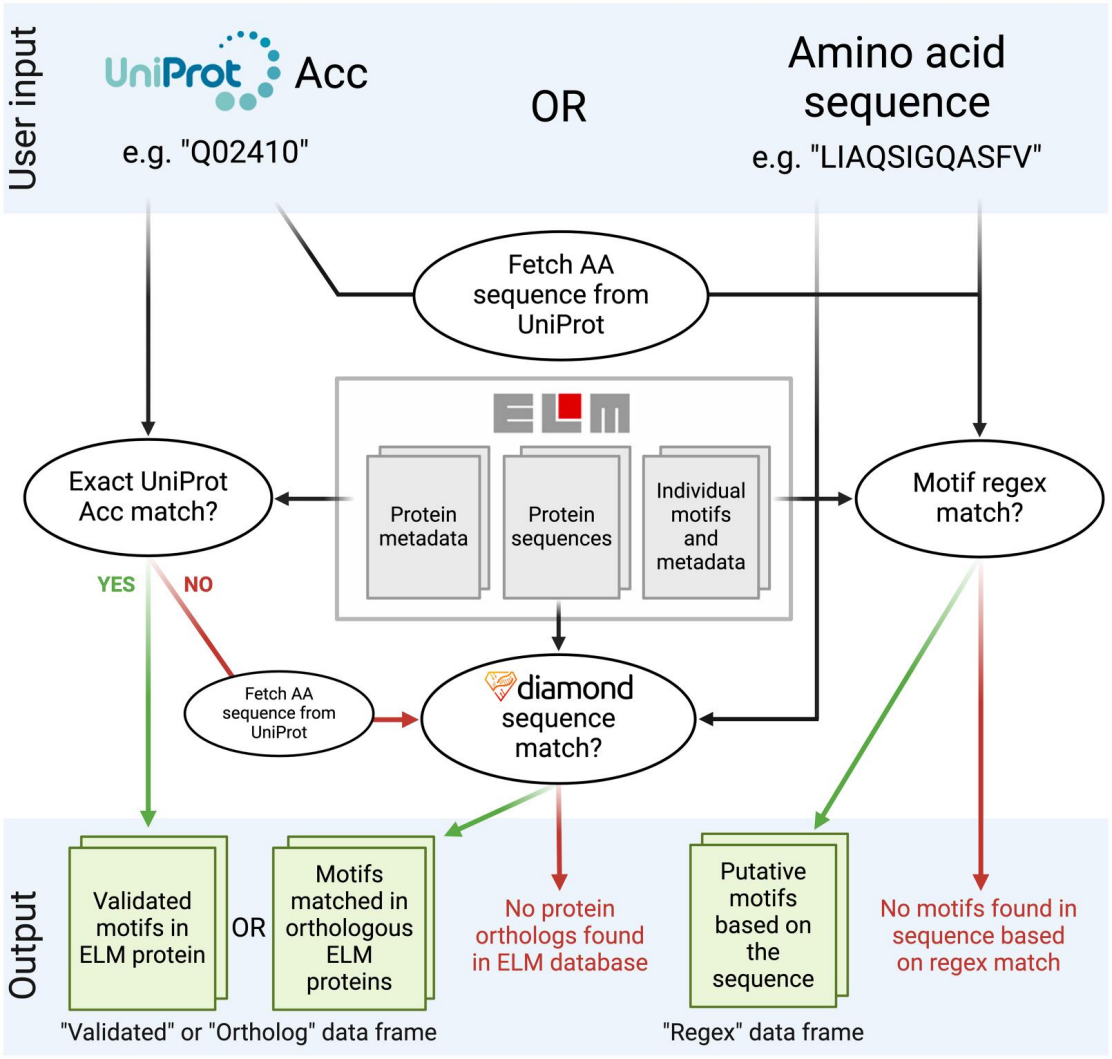
Schematic overview of the gget elm back-end.

Users can submit an amino acid sequence or a UniProt Accession to *gget elm. gget elm* captures both homology-based matches corresponding to curated motifs in orthologous proteins in the ELM database and POSIX regular expression (regex) matches corresponding to candidate motifs in the provided sequence. Hence, *gget elm* returns two separate data frames (or JSON formatted dictionaries for use from the command line) containing the respective motif matches and extensive information about each motif. Figure 2 provides an overview of the *gget elm* back-end.

After installing *gget* ($pip install gget), the user downloads the ELM database reference information using a specialized module, *gget setup*, with the command $gget setup elm. This command may be repeated at any time to update the local copy of the ELM database, which currently requires a total of 3 MB of disk space. The files are saved in the *gget* installation directory.

If the user submits a UniProt Accession to *gget elm* and the protein is not present in the ELM database, its amino acid sequence is fetched from UniProt (UniProt Consortium 2021). Using the DIAMOND alignment algorithm (Buchfink *et al.* 2021), the sequence is compared to the motif-containing proteins in the ELM database. *gget elm* returns all motifs associated with orthologous proteins, including information about each orthologous protein, and extensive details on each motif. *gget elm* also returns alignment scores for each DIAMOND hit, including identity and coverage percentages and Boolean output on whether the orthologous motif is contained within the overlapping region between the query and subject sequence. To compute the regex data frame, *gget elm* considers all regex expressions from the ELM database and scans them against the provided amino acid sequence to report all matches. The data from the ELM database are combined to return relevant information about each matched interaction motif, including motif description, type, sequence, location in the ortholog and query sequence, and host taxonomy, for both data frames. How different types of user input traverse the *gget elm* back-end is explored in this Google Colab notebook: https://tinyurl.com/4bd5h8hr.

*gget elm* builds on existing *gget* modules, such as *gget seq* to fetch amino acid sequences from UniProt, and a new module developed in parallel with *gget elm*: *gget diamon*d, which aligns sequences using the DIAMOND algorithm (Buchfink *et al.* 2021) and can be used independently from *gget elm*.

**Figure 1.**
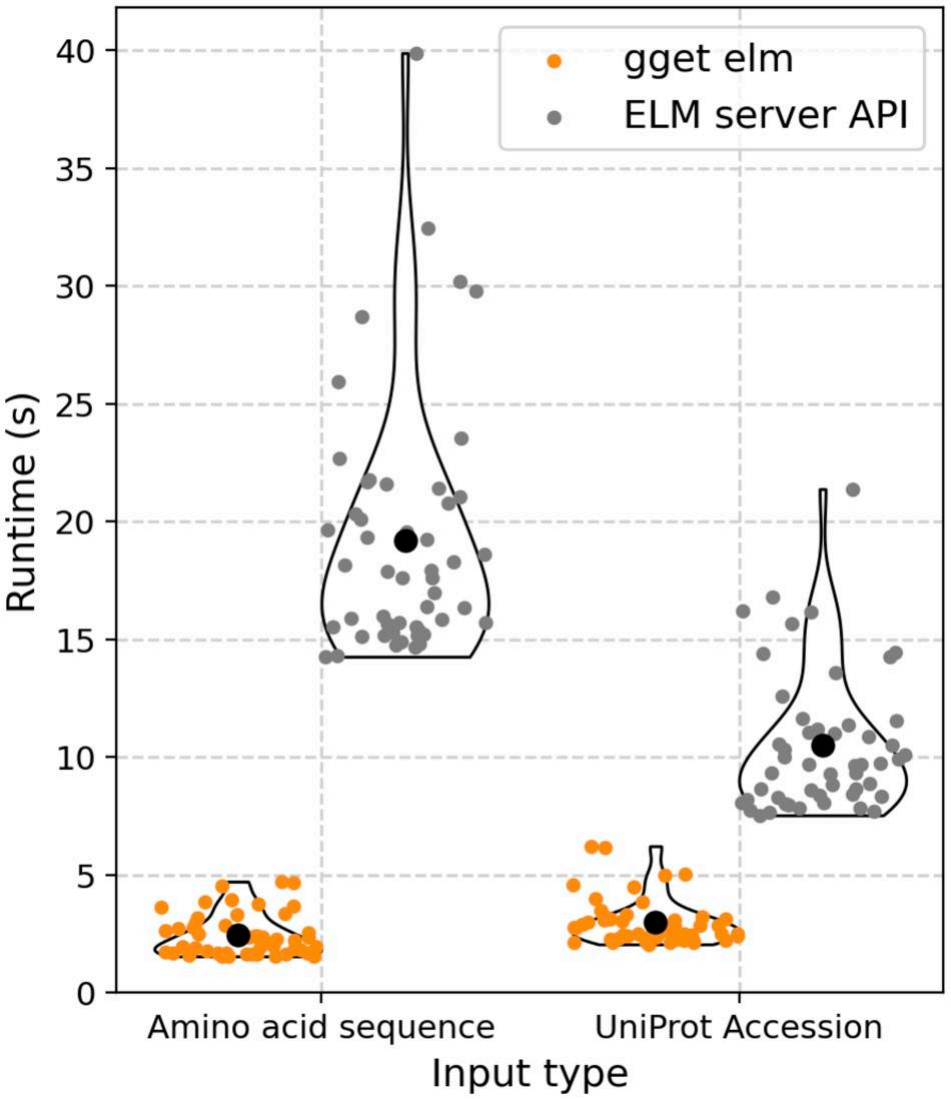
Runtime comparison for 50 amino acid sequences and 50 UniProt Accessions submitted to *gget elm* and the ELM server API. For the ELM server API, a 3-min wait time was observed between each request to comply with the server rules. These wait times were not taken into account when measuring the runtimes. The black dot denotes the mean. The code used to generate this figure can be found here: http://tinyurl.com/bdc6mhm3.

While *gget elm* results are similar to results obtained through the ELM web interface, they may not be identical due to differences underlying the computations. For example, *gget elm* uses DIAMOND for fast and sensitive local alignment of the amino acid sequences to identify orthologous proteins, whereas the ELM web interface has its own suite of back-end tools and deliberately limits the number of proteins in the output to be manageable for the web server (Chica *et al.* 2008). In a comparison between the “regex” data frame returned by *gget elm* and the results obtained through the ELM server API for 50 amino acid sequences and 50 UniProt Accessions, *gget elm* returned results 8× faster for amino acid sequences and 3.5× faster for UniProt Accessions on average (Fig. 1). For the ELM server API, runtimes are further increased significantly by a mandatory 1-min wait time between amino acid sequence requests, and a 3-min wait time between UniProt Accession requests to comply with the server usage recommendations and avoid 429 errors. The results returned by both methods matched 100% across all tested amino acid sequences and UniProt Accessions. The code to reproduce this analysis can be found here: http://tinyurl.com/bdc6mhm3.

## 3 Usage and documentation

Akin to all modules contained within *gget* (Luebbert and Pachter 2023), *gget elm* features an extensive manual available as function documentation in a Python environment or as standard output using the help flag [-h] in the command line. The accuracy of the returned results is maintained through extensive unit tests, which automatically run on a bi-weekly basis. The complete manual with examples can be viewed on the *gget* website in English (https://pachterlab.github.io/gget/en/elm) and in Spanish (https://pachterlab.github.io/gget/es/elm).

*gget* can be installed from PyPI using the command line with the following command:

$ pip install gget

Alternatively, *gget* can be installed using Anaconda:

$ conda install -c bioconda gget

Example *gget elm* commands to find ELMs in a protein from its amino acid sequence or UniProt Accession look as follows:

Command line (JSON formatted results are saved in a folder named “results”):

$ gget setup elm # Downloads/updates local ELM database

$ gget elm -o results LIAQSIGQASFV

$ gget elm -o results––uniprot Q02410

Python (two data frames are returned):

⋙ gget.setup(“elm”) # Downloads/updates local ELM database

⋙ ortholog_df, regex_df = gget.elm(“LIAQSIGQASFV”)

⋙ ortholog_df, regex_df = gget.elm(“Q02410”, uniprot=True)

The [––threads][-t] (Python: “threads”) argument can be used to multithread the sequence alignment for increased speed for large-scale computations. The following tutorial demonstrates how *gget elm* can be combined with the IUPred3 API (Erdős *et al.* 2021) to filter putative ELMs located within IDRs and thereby limiting false positive matches: http://tinyurl.com/mw5s5yf3.

## 4 Proof of concept

### 4.1 *gget elm* reports the loss of a protein interaction motif involved in DNA repair in a carcinogenic BRCA2 mutation

BReast CAncer gene 2 (BRCA2) plays an essential role in DNA repair through homologous recombination, and heterozygous germline defects in BRCA2 increase the risk of breast cancer. The promotion of homologous recombination by BRCA2 requires its association with the partner and localizer of BRCA2 (PALB2) (Hanenberg and Andreassen 2018). This important protein–protein interaction occurs at the site of a linear motif (ELM: LIG_PALB2_WD40_1, regex: [….WF.L]), which can be recognized by *gget elm*. We analyzed the wild-type BRCA2 sequence and a mutant BRCA2 sequence with a single amino acid substitution (W31C), previously described as carcinogenic due to a loss of interaction with PALB2 (Oliver *et al.* 2009). *gget elm* accurately reports the loss of the PALB2 interaction motif in the mutant sequence compared to the wild-type sequence: https://tinyurl.com/yc5r2b5m.

## Supplementary Material

btae095_Supplementary_Data
